# Characterization of small extracellular vesicles from ovarian cancer patients and pre-diagnostic patient samples: Evidence from the Danish blood donor study

**DOI:** 10.1371/journal.pone.0323529

**Published:** 2025-05-15

**Authors:** Nanna Lond Skov Frisk, Malene Møller Jørgensen, Rikke Bæk, Amila Iriskic Atic, Thorsten Rejkjær Brodersen, Sisse Rye Ostrowski, Margit Hørup Larsen, Dorthe Posselt, Estrid Høgdall, Claus Høgdall, Ole Birger Vesterager Pedersen, Louise Torp Dalgaard

**Affiliations:** 1 Department of Science and Environment, Roskilde University, Roskilde, Denmark; 2 Department of Clinical Immunology, Zealand University Hospital, Køge, Denmark; 3 Department of Clinical Immunology, Aalborg University Hospital, Aalborg, Denmark; 4 Department of Clinical Medicine, Aalborg University, Aalborg, Denmark; 5 Novo Nordisk A/S, Måløv, Copenhagen, Denmark; 6 Department of Clinical Immunology, Rigshospitalet, Copenhagen, Denmark; 7 Department of Clinical Medicine, Faculty of Health and Medical Science, University of Copenhagen, Copenhagen, Denmark; 8 Department of Pathology, Herlev and Gentofte Hospital, Copenhagen, Denmark; 9 Department of Gynaecology, Rigshospitalet, Copenhagen, Denmark; Nathan S Kline Institute, UNITED STATES OF AMERICA

## Abstract

**Aim:**

Ovarian cancer (OC) is the leading cause of gynecological cancer deaths. Current biomarkers of OC are not specific or sensitive enough. Extracellular vesicles (EVs), EV surface proteins and their cargo microRNA (miRNA) show potential as biomarkers. This study aimed to characterize the ability of EVs to identify early OC-biomarkers among blood donors six months before their diagnosis.

**Methods:**

Study groups of OC patients, benign tumor patients (B), healthy blood donors (Control), and blood donors with incident OC diagnosis within six months of the last blood draw (Pre-diagnostic; PD) were established. Small EVs were enriched from plasma using ultracentrifugation. EVs were characterized by Dynamic Light Scattering (DLS), EV Array, NanoFlow Cytometry, Nanoparticle Tracking Analysis, and Western blots. RNA from EVs was isolated. A discovery study was performed on OC and B patients using the TaqMan Array Human MicroRNA A card. A validation study of 9 specific miRNAs was performed using RT-qPCR.

**Results:**

With DLS, it was identified that the OC patients’ EVs were more heterogeneous in size compared to the other groups. Western blot identified CD63 and TSG101 in the EV enrichments. EV Array assessed 22 known protein biomarkers. TaqMan MicroRNA Array cards indicated a differential miRNA abundance between OC and B; however, technical replication and validation could not validate this pattern.

**Conclusion:**

This study has analyzed EVs in OC, B, Control, and PD women. More extensive investigations of EV CD9, CD151, and CD81 in conjunction with other risk factors and well-known biomarkers like CA125 or HE4 should be the main objectives of future research.

## Introduction

Ovarian cancer (OC) is the leading cause of gynecological cancer deaths in women worldwide [1]. Women often present with advanced-stage disease because OC has non-specific symptoms [2]. In Denmark, more than 70% of women diagnosed with OC have advanced-stage cancer at the time of the diagnosis, and only 42% of these women survive five years [1]. Cancer-antigen 125 (CA125) is the most common blood-based biomarker currently used; however, it is not very sensitive for early diagnosis, as only 50% of patients with stage I have elevated CA125 [3]. Furthermore, CA125 can be elevated due to other gynecological conditions [4]. Based on encouraging results, the US Food and Drug Administration approved using human epididymis 4 (HE4) in 2011. The key advantage of HE4 is that it is not increased in endometriosis, in contrast to CA125 [5]. The potential of HE4 has prompted the development of biomarker algorithms, such as the Risk of Ovarian Malignancy Algorithm (ROMA) and, more recently, the Copenhagen Index, which integrates HE4 and CA125 [6,7]. However, at present, not enough research is available to estimate how well HE4 can identify early-stage OC [8,9].

Extracellular vesicles (EVs) are double-membrane bound particles secreted by most cell types, including cancer cells [10], which are secreted to various body fluids [11,12]. EVs are of interest due to their potential as OC disease biomarkers [13] and can be subdivided into three main groups: small EVs (50–150nm), microvesicles (50–1000nm), and apoptotic vesicles (>1000nm) [14]. However, EVs can be of almost any size or diameter in a continuum, meaning that based on size alone, there are no distinguishable populations of small and large EVs. The size distribution of enriched EVs will depend on the chosen method(s) employed for separating or enriching the sample [15–17] and their biological origin. Studies find that different approaches will provide different EV preparations starting from the same material [16]. A proportion of EVs have an endosomal origin and are formed by exocytosis of multivesicular bodies, and are also termed exosomes. In contrast, other EVs are released by direct budding of the cell membrane into the extracellular space [13,18]. Small EVs are enriched in various biomolecules, e.g., proteins, lipids, and nucleic acids, such as microRNAs (miRNAs), for which the content reflects the EV origin [19] and where the EV membrane protects the cargo from degradation [20]. MiRNAs are a class of small non-coding (nc)RNAs that have an average of 22 nucleotides in length [21] often carried by EVs. MiRNAs and EVs play a role in cell-to-cell communication [22] and both can be isolated from body fluids such as ascites, saliva, urine, and blood. Moreover, EVs have been shown to mediate the transfer of the specific cargo of the EVs between organs and tissues, in addition to cell-to-cell communication [23]. Cancer cells are suggested to release considerably more EVs with a different composition than normal cells [24,25].

Therefore, new biomarkers with a higher sensitivity for early-stage OC are essential to promote earlier diagnosis and treatment for women with OC. A non-invasive blood test may be a clinically beneficial solution to screen women for OC, and to that end, EVs and their cargo may be advantageous. EVs could be carrying specific signatures reflecting cancerous growth. This study aims to characterize and investigate the ability of EVs to identify undiagnosed OC among otherwise healthy blood donors prior to OC diagnosis from other relevant patients and control groups.

## Materials and methods

### Patients and plasma sample preparations

Plasma samples originated from the nationwide Danish Cancer Biobank (DCB), Herlev [26], and the nationwide Danish Blood Donor Study [27,28]. The current study consists of 12 late-stage OC patients, 12 patients with benign tumors, 12 healthy blood donors, and 12 blood donors with incident OC diagnosis within 6 months of the last blood draw. The medical laboratory technologist collected the Danish Blood Donor Study samples in the blood donor centers. Venous blood from the Danish blood donor study was collected in gel ethylenediaminetetraacetic acid (EDTA) tubes; the samples were centrifuged for 10 min at 2000 g, 4°C. The EDTA tubes were frozen at -20°C until all samples were collected. The EDTA tubes were thawed, and the plasma phase was separated into 1.5mL microcentrifuge tubes and stored at -20°C. The EDTA plasma samples from the DCB were collected prospectively from patients within 2 weeks of the operation. The samples were centrifuged at 2,000 g for 10 min within 2h of collection, and plasma separated. After centrifugation, samples were aliquoted and stored at -80°C until analysis. The samples were collected in a biobank in September 2020.

### Ethics and transparency

This study was approved by the local Scientific Ethical Committee of Region Zealand and the Capital Region, Denmark (approval no. SJ-740, SJ579, H-15020061), and the Data Protection Agency in the Capital Region and local ethics committees approved the study (approved no. P-2019–99, P-2019–656). The study was performed according to the Declaration of Helsinki II. All subjects gave written informed consent prior to inclusion, and no individual participants could be identified during or after data collection. The EV characteristics of the study were submitted to EV-TRACK under the first author (Frisk, Nanna) and a completed MIBLOOD-EV checklist enclosed as Supporting information.

### Extracellular vesicle enrichment and characterization

The EVs were enriched using a Hitachi CP100NX ultracentrifuge (Hitachi Koki, Tokyo, Japan) using rotor T-1270. Plasma samples were initially centrifuged at 2000 × g for 30 min to remove large cell debris and thrombocytes. The supernatant was collected and centrifuged at 12000 × g for 45 minutes to remove large EVs and smaller debris. The supernatant was carefully removed and centrifuged twice at 100,000 × g for 120 min. The enriched EVs were re-suspended to 1mL 0.02μm filtered PBS and were stored at 5°C until further use. Dynamic Light Scattering (DLS) was conducted using a Zetasizer (Malvern Panalytical Malvern, UK) to estimate EV size and calculate the polydispersity index (PDI). Nanoparticle tracking analysis (NTA) was performed according to the manufacturer’s instructions with the ZetaView PMX-230 TWIN Laser system and the software ZetaView version 8.05.16 SP7 (Particle Metrix, Inning, Germany). The measurements were collected at 11 separate positions using a 1:1000 dilution with 0.2µm PBS. The data acquisition settings were adjusted to 85% sensitivity, a shutter of 200, and a rate of 30 frames per second. Histogram analysis was based on recorded tracings between 15nm and 500nm, and traces were analyzed in bins of 15nm (default instrument settings). Nano Flow cytometry (nFCM) was performed according to the manufacturer’s instructions with the NanoAnalyzer instrument (NanoFCM, Nottingham, United Kingdom) using standard settings. Transmission electron microscopy was performed at the Core Facility for Integrated Microscopy at the University of Copenhagen. Samples were stained with uranyl acetate and examined with a Philips CM 100 transmission electron microscope operated at an accelerating voltage of 80 kV and equipped with a SIS MegaView II camera. Digital images were recorded with the analySIS software package

### Immunoblot analysis

The protein concentration of re-suspended EVs was assessed using the Pierce BCA Protein Assay Kit (ThermoFisher Scientific, Waltham, Massachusetts, USA) per the manufacturer’s instructions, and Western blotting through a 12% bis-tris SDS-page (ThermoFisher Scientific, Waltham, Massachusetts, USA) employed The following antibodies were used: Albumin (diluted 1:2000) (HYB-192–01, SSI, Copenhagen, Denmark), TSG101 (diluted 1:1000) (BD 612696, BD Biosciences, Lyngby, Denmark, Cambridge, UK), and CD63 (1:1000) (Ab8219, Abcam, Cambridge, UK). The positive control (PC) was thrombocyte lysate. We tested, by Western blot for albumin and CD63, pooled plasma samples from anonymous blood donors during optimization of the EV enrichment procedure. Similarly, Following EV enrichment of biobank samples, selected patient and blood donor samples were tested by Western blot for CD63 and TSG101.

### EV Array analysis of protein surface markers

Enriched EV samples were applied to an EV Array based on antibody microarray. The array was prepared on epoxysilane-coated slides (75.6mm x 25.0 mm; SCHOTT Nexterion, DE) using microarray printer sciFLEXARRAYER S12 using a size 60 piezo capillary with type 3 coating (Scienion GMBH, DE). Temperature and humidity were kept stable throughout the experiment. A selection of 22 antibodies were printed in triplicates at 200 µg/ml in PBS with 50mM Trehalose (S1 Table). Positive control was biotinylated human IgG (100µg/mL), and negative control was PBS with 50mM Trehalose [29–31]. The EV Array was prepared as described by Jørgensen et al., 2013 [29] with modifications: In short, the microarray slides were initially blocked (50 mM ethanolamine, 100 mM Tris, 0.1% SDS, pH 9.0) prior to incubation with 50 µ L of enriched EVs in incubation buffer (0.2% Tween®20, 0.5X Casein (B6429, Sigma-Aldrich) in PBS). The incubation was performed in Multi-Well Hybridization Cassettes (ArrayIt Corporation) at RT for two hours followed by overnight incubation at 4 °C. Following a short wash (0.2% Tween®20 in PBS), the slides were incubated with a cocktail of biotinylated detection antibodies (anti-human-CD9, -CD63 and -CD81, LifeSpan BioSciences, WA, USA) diluted 1:1500 in incubation-buffer. After a wash, a subsequent 30-minute incubation step with Cy5-labelled streptavidin (Life Technologies) diluted 1:1500 in the wash buffer was carried out for detection. Prior to scanning, the slides were washed in a wash-buffer, then in ultrapure/deionized water, and finally dried using a Microarray High-Speed Centrifuge (Array It Corporation). Scanning and spot detection was performed as previously described Jørgensen et al. [31].

### Extracellular vesicle miRNA analysis

Total RNA was isolated from 250 µ L EV suspension using TriReagent LS (Sigma-Aldrich, St. Louis, Missouri, USA) according to the manufacturers’ protocol, with the addition of glycogen (5 µg per 250 µ L plasma) as a coprecipitation product and dissolved in 20μL DEPC treated MilliQ water. RNA concentration and purity was determined by NanoDrop ND-100 spectrophotometer (ThermoFisher Scientific, Waltham, Massachusetts, USA). The RNA samples of OC and B were first used for a PCR-based array discovery study, followed by a technical replication and validation study where OC, B, Control, and PD were used. The discovery study used MegaPlex RT reaction with MegaPlex RT primers (ThermoFisher Scientific, Waltham, Massachusetts, USA) to create cDNA. The cDNA was pre-amplified using the Preamp Master Mix 2X and MegaPlex preamp primers according to the manufacturer protocol (ThermoFisher Scientific, Waltham, Massachusetts, USA). QuantitativePCR (qPCR) was performed using the TaqMan Universal PCR Master mic (2X) (ThermoFisher Scientific, Waltham, Massachusetts, USA) and the TaqMan Array Human MicroRNA A card set v3.0 (ThermoFisher Scientfic, Waltham, Massachusetts, USA), consisting of 377 miRNAs commonly observed in circulation and 7 control wells. The qPCR was run on ViiA 7 Real-Time PCR (ThermoFisher Scientific, Waltham, Massachusetts, USA). A global mean of the samples was used as a normalization for the array card qPCR data analysis.

For the technical replication and validation of the miRNAs identified in the miRNA arrays, cDNA synthesis was performed using the TaqMan microRNA Reverse Transcription kit using an input of 100ng RNA (ThermoFisher Scientific, Waltham, Massachusetts, USA). The cDNA was diluted 1:10 in sterile water and stored at -20°C. Diluted cDNA was mixed with appropriate miRNA primers and PowerUp SYBR Green Master Mix (ThermoFisher Scientific, Waltham, MA, USA) [32,33]. All qPCR assays were performed in duplicates, analyzed using the Lightcycler 96 instrument (Roche LifeScience, Basel, Schwitzerland) and calibrated using an interplate-calibrator consisting of pooled sample material. Raw CT-values were quantified using the standard curve method and normalized against the geometric mean of two reference genes: miR-486-5p and U6 (which were unchanged between groups in the discovery study and the validation study). The oligonucleotide sequences used are listed in S2 Table.

### Statistical analysis

Continuous variables were tested for normality of distribution using the Shapiro-Wilks test and log-transformed if skewed. Variables with approximately normal distribution are presented as mean ± standard deviation (SD), then those with skewed distribution are presented as median with interquartile range. We used ANOVA with Tukey’s test for multiple comparisons when variables were normally distributed or the pair-wise Mann-Whitney U-test when variables were not normally distributed (following logarithmic transformation). A *p*-value of < 0.05, following adjustment for multiple testing, was considered statistically significant. All analyses were conducted using SPSS v. 28.0 (SPSS Inc., Chicago, IL, USA). Graphical representation was done in GraphPad PRISM version 10.0 (GraphPad Inc., La Jolla, CA, USA) or R-studio version 2024.04.2 and R version 4.3.1 using the pheatmap package [34].

## Results

**Fig 1:**
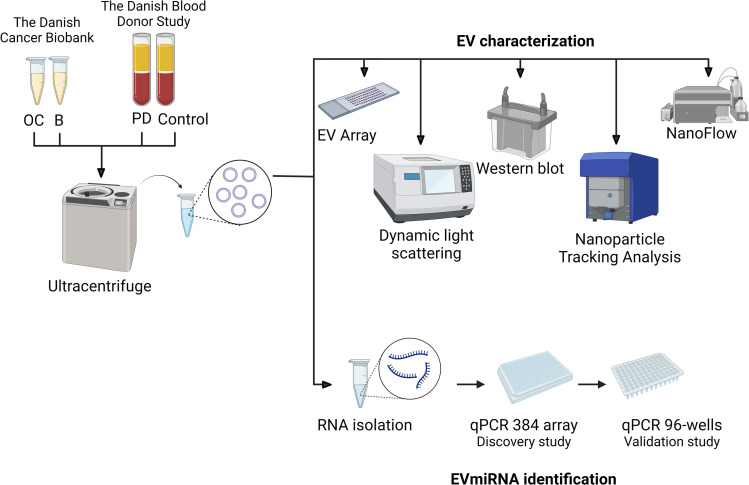
Flowchart of study. EVs were enriched using an ultracentrifuge. The EVs were characterized using Western blot, dynamic light scattering, nanoflow, nanoparticle tracking analysis, and EV Array. The EV-bound miRNAs were assessed using qPCR 384-Array in the discovery study, followed by a technical replication and validation study in single-target 96-well qPCR.

In this study, we investigated biomarkers for OC. We enriched EVs, which we characterized using Western blots, DLS, NTA, nFCM, and an antibody array for EV markers. We also isolated total RNA from the EVs, which we used for a discovery qPCR array study on the OC and B samples, followed by a technical replication and a validation qPCR study using OC, B, Control, and PD samples (Fig 1).

### Characteristics of study population

This study evaluated EV preparations of plasma samples from the DCB, containing plasma from women with high-grade serous OC (OC) and women with benign gynecological tumors (B). The DBDS contains plasma from healthy blood donors (Control) and blood donors with incident OC diagnosed shortly after blood donation (prediagnosis; PD). Twelve women were included in each group (Table 1), and groups were matched by age. The DBDS samples had BMI and smoking status available, whereas the OC women had cancer antigen 125 (CA125) measured and Risk of Malignancy (RMI) calculated [35], BMI and FIGO stages available (Table 1).

**Table 1:** Baseline characteristics for clinical parameters of study participants measured at inclusion.

	Ovarian cancer (n = 12)	Benign (n = 12)	Control (n = 12)	Pre-diagnosis (n = 12)	P-value
**Age** (years)	63 ± 12	59 ± 12	52 ± 17	56 ± 10	NS
**BMI** (kg/m^2^)	25 ± 3.4	–	24 ± 4.4	24 ± 3.2	NS
**CA125** (kU/L)	1874 ± 1071	–	–	–	–
**RMI**	12941 ± 10774	–	–	–	–
**Stage**: IIIC; Iva; IVb; IVc	6; 3; 3; 0	–	–	–	–
**Smoking**: Former; present; non-smoker, missing	–	–	5; 0; 4; 3	2; 4; 4; 1	–

Data are presented as mean ± standard deviations. Continuous data were analyzed by one-way ANOVA with Tukey’s post-hoc test for multiple comparisons. NS, not significant. BMI, body mass index. CA125, Cancer-antigen 125. RMI, Risk of malignancy index.

### Extracellular vesicle characteristics

**Fig 2:**
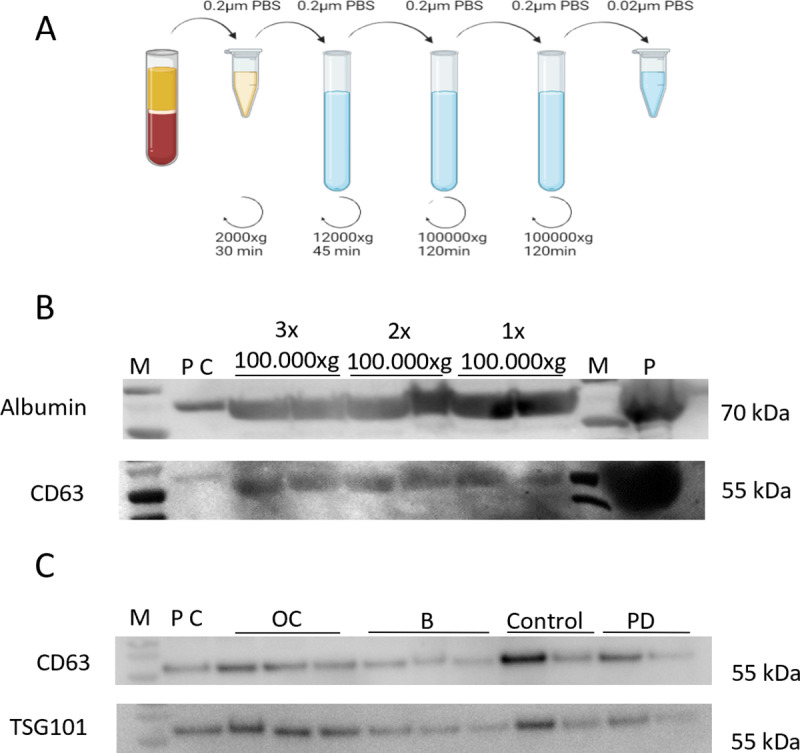
EV enrichment method and Western blot characterization. A. A schematic overview of the final EV enrichment procedure using ultracentrifugation. B. Western blots during optimization of EV enrichment, a test of albumin, and CD63 using pooled plasma samples from anonyomous blood donors (P: Plasma, M: Molecular marker, PC: Positive control; reticulocyte lysate). **C.** Identification of EV markers CD63 and TSG101 in selected patient samples (sample numbers: OC 24, 27, 50; B 27, 28, 29; Ctrl 2, 13, PD 3, 6). (M: Molecular marker, PC: Positive control; reticulocyte lysate).

Due to considerable albumin amounts in initial EV preparations, we optimized the EV enrichment protocol: We tested if washing and several centrifugations at 100,000 × g would decrease the albumin load. The standard reported protocol for EV preparation by ultracentrifugation has one centrifugation at 100,000 × g (2h) [36]; and we washed the pellet after the first centrifugation followed by a second 100,000 × g spin (2h) (Fig 2A), with a third time repeat. The enriched EVs were used for Western blot analysis for albumin. The amount of albumin declined following additional wash and centrifugation steps (Fig 2B). Furthermore, CD63 was tested to demonstrate the presence of EVs (Fig 2B). As a compromise between EV yield and albumin load reduction, the final protocol included one extra wash and centrifugation step at 100,000 × g for a total of 2 times ultracentrifugation at 100,000 × g to minimize albumin in the patient samples, while preserving levels of EVs, measured by CD63 levels. EV preparations were finally adjusted to 1mL per sample. A selection of patient EV samples were tested using Western blotting, and all samples were positive for CD63 and TSG101 (Fig 2C). Furthermore, we tested a small selection of samples by transmission electron microscopy and verified that EVs were present, although images also indicated the presence of some contaminations (S1 Fig).

Characterization of the enriched EVs included DLS, NTA and nano FCM:as well as yield of RNA and protein. The overall concentrations of particles detected by each method were in the same range (10^9^ to 10^10^ particles/mL). The concentrations of RNA ranged from 9-15 ng/μL (Table 2), and the protein concentrations ranged from 0.5-0.6 μg/μL, with no significant differences between groups. DLS and NTA were used to identify the size (diameter) of the enriched EVs (Table 2), with the measured sizes having ranges of 99.1-114.5nm for DLS and 132.7-138.3nm for NTA, consistent with EVs being a major particle contributor to these measurements.

**Table 2:** *C*haracterization of EVs.

	Ovarian cancer (n = 12)	Benign (n = 12)	Control (n = 12)	Pre-diagnosis (n = 12)	p-value
RNA isolated (ng/μL)	9.2 ± 5.3	12.1 ± 8.0	14.7 ± 9.0	10.1 ± 4.8	^NS^
Protein isolated (μg/μL)	0.5 ± 0.12	0.5 ± 0.14	0.6 ± 0.18	0.6 ± 0.1	^NS^
DLS (nm)	114 ± 149	111 ± 62	104 ± 29	99 ± 17	^NS^
PDI	0.4 ± 0.12	0.4 ± 0.09	0.3 ± 0.06	0.3 ± 0.03	^NS^
NTA (particles/mL)	4.4 ⋅ 10^10^ ± 5.3 ⋅ 10^10^	3.9 ⋅ 10^10^ ± 4.9 ⋅ 10^10^	3.9 ⋅ 10^10^ ± 1.9 ⋅ 10^10^	3.2 ⋅ 10^10^ ± 1.8 ⋅ 10^10^	^NS^
NTA Particle size (nm)	138 ± 34	139 ± 12	132 ± 18	135 ± 10	^NS^
NanoFlow (particles/mL)	3.3 ⋅ 10^9^ ± 4.4 ⋅ 10^9^	9.7 ⋅ 10^9^ ± 1.7 ⋅ 10^10^	6.9 ⋅ 10^10^ ± 9.4 ⋅ 10^10^	3.9 ⋅ 10^10^ ± 4.6 ⋅ 10^10^	^#^

Data are presented as mean ± standard deviations. EV, extracellular vesicles. PDI, Polydispersity index. Continuous data were analyzed using ANOVA, followed by Tukey’s post hoc test. ^#^ p-value = 0.022 ovarian cancer vs. control.

**Fig 3:**
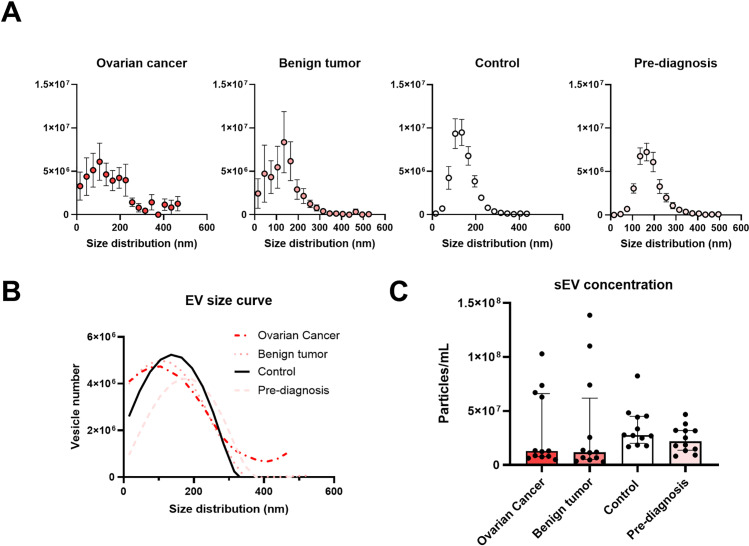
EV size distribution determined using NTA. A. The frequency of particles and their size distribution measured by NTA for Ovarian cancer, Benign tumor, Control and Pre-diagnosis samples. Shown is mean frequency ± SEM, n = 12/group. B. Smothened curves of the data in Fig 3A plotted together indicating a wider size distribution for Ovarian cancer and Benign tumor samples (2^nd^ order smoothing, 7 neighbors). C Violin plots of sEV concentrations (based on particles sizes 15-195nm) for Ovarian cancer, Benign tumor, Control and Pre-diagnosis samples (n = 12/group).

DLS measurements of EV size were lower than for NTA, which could be due to small EVs being measured with lower efficiency by NTA [37]. EVs from the OC and B samples were more heterogeneous in size with larger SD and polydispersity index (PDI), although this did not significantly change between groups. The concentration of particles was measured using NTA and nFCM: While NTA did not detect differences between groups, the nFCM counted fewer particles in EVs from OC compared with Ctrl (P < 0.05)(Table 2). NTA has a lower limit of detection (15nm) compared with nFCM (~40–70nm) [38], which might account for this difference in particle counts, if the size distributions of EVs of OC and B samples were different from Ctrl and PD samples. However, when we extracted size distributions from NTA-measurements (Fig 3) and plotted the number of observed particles from NTA analysis as a function of size (in nm) for the four groups (Ctrl, PD, OC and B) (Fig 3A), it was not possible to identify differences in vesicle abundances between the groups by NTA, as indicated by nFCM, which is supported by similar protein concentrations between groups (Table 2). However, the plots (Fig 3A) showed that OC and B samples had a more dispersed distribution of particles compared to Ctrl and PD samples (Fig 3B), as well as higher variation between individual samples with regard to size (Fig 3A) and concentration (Fig 3C). The higher spread in particle size distribution of OC and B samples determined from NTA histograms is consistent with the marginally increased PDI determined by DLS as well as slightly lower particles counts by nFCM. Especially particles in the size range of 100–200nm appeared slightly less abundant in OC samples compared with Ctrl (and PD) samples: Median numbers for particles sizes 105nm (OC: 2.6 ⋅ 10^6^ vs. 6.3 ⋅ 10^6^, p = 0.16), 135nm (OC: 3.0 ⋅ 10^6^ vs. 7.8 ⋅ 10^6^, p = 0.03), 165nm (OC: 2.9 ⋅ 10^6^ vs. 6.5 ⋅ 10^6^, p = 0.13) and 195nm (OC: 4.3 ⋅ 10^6^ vs. 6.1 ⋅ 10^6^, p = 0.10), however when corrected for four multiple tests using Bonferroni, the individual differences were not significant.

### Protein biomarkers measured using the EV Array

**Fig 4:**
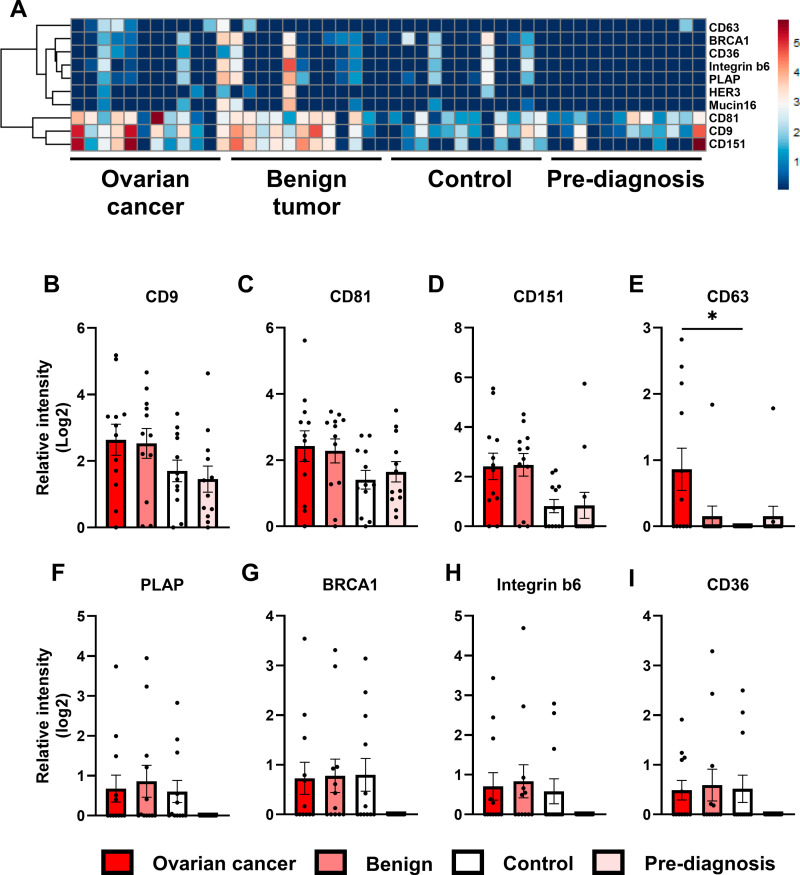
Protein biomarkers tested using the EV Array. A. Heatmap drawn with Euclidian clustering was based on robustly detected protein biomarkers in enriched EV samples; detected in at least 6 samples. Missing data are given a 0.0000001 value resulting in dark blue. B-I Individual sample level presentation of levels of protein biomarkers identified in the four groups: OC, B, Ctrl, and PD, depicted as relative mean intensity with standard deviation (SD) (a.u.). B. CD9, C. CD151, D. CD81, E. CD63, F. PLAP, G. BRCA1, H. Integrin b6, I. CD36. Statistical comparisons between groups were made using one-way ANOVA on logarithmic transformed data with Tukey’s test for multiple comparisons. *: P < 0.05.

An EV Array was performed on enriched EV samples. Twenty-two biomarkers were tested: Different vesicle biomarkers, cancer biomarkers, and tissue markers (for the total list, please refer to S3 Table). The identification of CD9 and CD81 on all EV preparations indicated the presence of EVs (Fig 4B, C). Furthermore, CD151 was more abundant in the OC and B groups, compared with Ctrl and PD samples (Fig 4D, p < 0.01 by two-way ANOVA), but was not different between any single group. EV CD63 levels were increased in OC samples compared with Ctrl (p < 0.05), but not significantly different in other comparisons (Fig 4E). Levels of PLAP, BRCA1, Integrin b6 or CD36 in EV preparations were not significantly different between groups (Fig 4F-I), but interestingly, for these four protein markers, levels were undetectable in PD samples. Overall, clustering indicated that EV markers such as CD9, CD81, and CD63 were more abundant in EVs from OC and B patients compared to Ctrl and PD samples. However, it was impossible to uniquely detect PD samples based on a pattern of EV markers.

Interestingly, Mucin16 (CA125) was only identified in two Ctrl samples and no PD samples and was only identified in three OC samples and three B samples (Fig 4A, S2Fig. A). The same pattern was identified for CD36: three OC samples, five B samples, three Ctrl samples, and zero PD samples detected CD36 on EVs (Fig 4A, I), though the positive samples were not the same as for Mucin16, indicating heterogeneity among the EV composition of individual patient samples. CEA and MUC1 were detected in one OC EV sample (S2 Fig C, D), while HER3 was not identified in Ctrl or PD samples and was only present at low levels in OC EV samples and higher levels in B EV samples (S2 Fig D). Ten of the tested markers were not measurable on EVs (Annexin V, LAMP2, Tspan8, CA9, HER2, HER4, TSG101, CD19–9, ALIX and EGFR). None of the tested EV protein biomarkers significantly differed between PD and Control samples and were regulated in the same direction in OC and PD samples.

### Identification of EVmicroRNAs having possible associations with ovarian cancer

**Fig 5:**
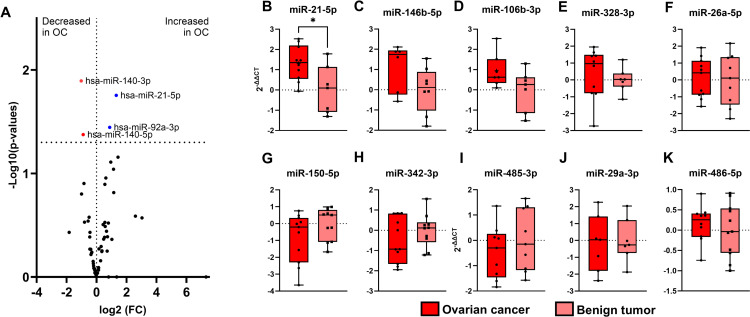
EV miRNA levels in OC vs. B samples based on Taqman MicroRNA Array Cards A. Volcano plot showing the fold-change between EV miRNAs in OC vs B groups and corresponding significance displayed as -Log10(p-value). B-G shows the TaqMan MicroRNA Array results, with 2ΔΔCT; the data are presented as mean ± SD. B. miR-21-5p. C. miR-146b-5p. D. miR-106b-3p. E. miR-328-3p, F. miR-26a-5p, G. miR-150-5p. H. miR-342-3p. I. miR-485-3p. J. miR-29a-3p, K. miR-486-5p. OC, ovarian cancer. B, benign tumor. * p < 0.05.

As a discovery study, 377 mature EVmiRNAs were investigated in OC (n = 12) and B (n = 12) samples using TaqMan microRNA array cards. Sixty-two miRNAs were robustly detected (defined as the EVmiRNA being identified in at least 6 samples in either OC or B). Twenty-one different EVmiRNAs appeared lower in OC and thirty-six different EV miRNAs appeared to be higher in OC compared with B patients (Fig 5A). Of the 21 decreased miRNAs, two significantly decreased: hsa-miR-140-5p and miR-140-3p (nominal p-values <0.05). Two EVmiRNAs were significantly increased in OC: hsa-miR-21-5p and hsa-miR-92a-3p. However, after correction for multiple testing, none of these miRNAs were significantly changed in OC compared with B tumor cyst patients (S3 Table). Nine different EVmiRNAs (hsa-miR-21-5p, hsa-miR-146b-5p, hsa-miR-106b-3p, hsa-miR-328-3p, hsa-miR-26a-5p, hsa-miR-150-5p, hsa-miR-342-3p, hsa-miR-29a-3p, hsa-miR-485-3p)(Fig 5B-K) were selected for technical replication and validation in further samples, Hsa-miR-21-5p was selected because it was nominally significantly increased in OC (Fig 5B). Hsa-miR-146b-5p (Fig 5C), -106b-3p (Fig 5D), and -328-3p (Fig 5E) were selected, because they showed a tendency for increased levels in OC women compared to B women. Hsa-miR-328-3p (Fig 5 E) and -26a-5p (Fig 5F) were selected because they were identified in our systematic review [39]. Hsa-miR-150-5p (Fig 5G), -342-3p (Fig 5H), and -485-3p (Fig 5I) were selected because their patterns between groups, even if not significant, showed that the EVmiRNAs tended to be less abundant in OC than in B women. Hsa-miR-29a-3p (Fig 5J) was selected because it has a known association with polycystic ovary syndrome (PCOS)) and is present in follicular fluid [40].U6 and hsa-miR-486-5p were selected as references for rt-qPCR (Fig 5K).

### Further investigation of EVmiRNAs in OC

**Fig 6:**
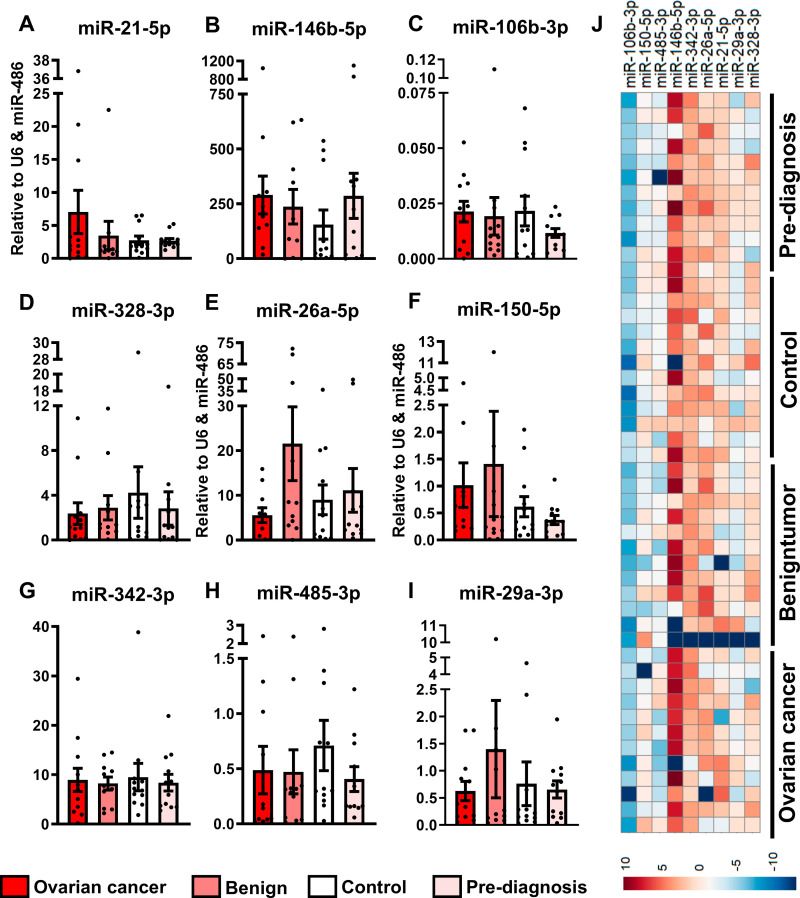
Technical replication and validation of EVmiRNAs. A. miR-21-5p. B. miR-146b-5p. C. miR-106b-3p. D. miR-328-3p, E. miR-26a-5p, F. miR-150-5p, G. miR-342-3p. H. miR-485-3p, I. miR-29a-3p. The data were normalized using U6 and miR-486 and presented as mean ± SD. J. Heatmap of the 9 miRNAs clustered using Euclidean clustering with the pheatmap R-package.

The nine selected EV miRNAs were assayed in a single RT-qPCR analysis in a technical replication and validation study (miR-21-5p, miR-146b-5p, miR-106b-3p, miR-328-3p, miR-26a-5p, miR-150-5p, miR-342-3p, miR-485-3p, and miR-29a-3p (Fig. 6 A-I). None of the EV miRNAs showed statistically significant differences between groups (Fig 6A-I). However, some of the same tendencies from the discovery study were observed: We observed tendencies of increased levels of miR-21-5p in OC women compared with the other groups, but the differences were not significant (Fig 6A).

When analyzing EV miRNA clustering in patient samples, eight of the nine EVmiRNAs formed one closely related cluster, whereas hsa-miR-146b-5p did not cluster with the others. Clustering to detect groupwise associations did not reveal a clear pattern (Fig 6J).

## Discussion

In the current study, we enriched, characterized, and verified EVs from plasma samples from OC patients and PD women and relevant controls (benign tumors and control subjects). A high abundance of CD9 and CD81-positive EVs was identified in the enriched EVs. Furthermore, 25 biomarkers were tested in enriched EV samples. The biomarkers combined EV markers, cancer antigens, and tissue specificity markers. The EV Array demonstrated the presence of EVs, as they were positive for CD9 and CD81. However, the EV Array did not indicate the presence of ALIX and TSG101. Western blots of selected patient samples were positive for TSG101 using the enriched EVs, perhaps indicating a difference in sensitivity between methods or different affinities between the antibodies used. The multi-analytical EV Array may need to be optimized to detect TSG101 reliably. CD9 and CD81 were measurable in all EV samples; however, CD63 was not identified in the Control samples, and in only 2 out of 12 Pre-diagnostic samples. Although there was heterogeneity with regard to detection of CD63 in EV samples, the levels of EV63 were significantly increased in OC samples compared with the Ctrl group (Fig 4E). In general, the EV levels of CD9, CD81 and CD151 were increased in OC and B samples compared with Ctrl and PD samples (Fig 4 B-D), which could be due to samples being obtained from two different biobanks, but increased levels of EVs have previously been reported in cancer patients [24,25].

Further characterization of EV preparations included DLS, NTA and nFCM methods, which all detected EV sizes within the expected size range. However, there were systematic differences between these methods: The NTA estimated EVs of a larger size than the DLS; this could be because the NTA measurements were performed later than the DLS measurements, and the samples were stored at 4°C, but it could also be due to the lower efficiency of the NTA in capturing small size EVs, due to their translucent nature and measurements in diluted samples [37]. We observed that OC and B samples had larger standard deviations for size for DLS data compared with Ctrl and PD samples, and similarly for OC samples for NTA data, indicating higher EV heterogeneity for these groups, which was in concordance with the observation of increasedPDI for OC and B samples. We further estimated the size distribution of detected particles based on NTA data, which also showed an increased spread in the EV size distribution in OC and B samples compared with Ctrl and PD samples (Fig 3). The nFCM and NTA identified similar particle quantities between groups, with the exception for the nFCM measurements between Controls and OC, which showed fewer counts by nFCM in the OC group. This could perhaps be explained by the more distributed size range of EVs in OC samples, leaving a higher fraction of OC sEVs uncounted by the nFCM method, whose lower size level of detection is above that of the NTA method [38]. There could be several explanations for the observed wider spread in sizes of isolated EVs from OC and B samples compared with Ctrl and PD samples: Although the sample storage times were matched, the samples from the cancer biobank were stored at -80°C, while the DBDS samples were stored at -20°C. Another explanation could be that cancer patients were previously observed to have an altered composition of EVs [24,25]. During the ultracentrifugation-based EV enrichment procedure, samples from all four groups were processed in parallel in smaller batches, thus, we do not believe the observation of increased size range in OC and B samples to be due to the sample work-up procedures.

We further investigated EV miRNAs for their potential as a novel biomarker category for OC. The discovery results indicated that EV miRNAs may potentially identify OC from B although only few miRNAs were potentially different between the two groups and none after adjustment for multiple testing (Fig 5). However, the technical replication and validation study did not conclusively support these results (Fig 6). Even though we did not identify statistically significant differences in EV miRNA levels, they may still have potential in early diagnostics. The small cohorts may affect the results because of the heterogeneity of samples. Thus, it is important that larger studies are carried out using well-characterized protocols and EV populations. Nonetheless, we suggest that a biomarker combining CA125, HE4, sonography results, menopausal status, and EVmiRNAs or CD9, CD81, and CD151 may have the potential to be used as a novel diagnostic tool for OC, although further studies are warranted. While growing evidence suggests that EVs reflect biological functions, the clinical use of EVs as biomarkers is thus far limited [41]. Research efforts until now have almost exclusively focused on developing and validating tests to aid in the assessment of OC, primarily targeting soluble biomarkers like CA125, HE4, or CEA, either alone or in combination with other tests such as imaging and risk factors like menopausal status. (RMI) [35,42,43].

The strength of the current study lies in the design of the study in which we have acquired pre-diagnostic samples from the DBDS biobank from women giving blood donations few months prior to their diagnosis with OC, and compared with samples from matched women who remained healthy, or from women with OC or having a benign ovarian tumor. Another strength lies in the comprehensive characterization of the EV enriched samples including multiple methods such as DLS, NTA, nFCM, electron microscopy, immunoblotting, EV array and microRNA determinations.

However, the current study also has a number of limitations: One limitation in the experimental design is due to the origin of samples from two separate biobanks: One of established OC with comparison samples having benign tumors, and the other containing pre-diagnostic samples from women being diagnosed with OC within 6 months compared with healthy, matched controls. The patients and controls were representative of the Danish population and the participants were age-matched, samples were collected using methods established by the Danish Regions Bio- and Genome Bank (RBGB). Both biobanks centrifuged the EDTA-gel glass samples within two hours of collection; the protocol instructed a centrifuge stage at 2000 g for 10 minutes. However, the samples were stored under slightly different conditions: While the DBDS samples were stored in the EDTA-gel glass at -20°C, the DCB EDTA samples were aliquoted into smaller tubes and stored at -80°C. Studies suggest the storage method for plasma plays a role in the particle size and integrity of the EVs [44,45]. Moreover, for further studies, experimental approaches with increased yield of EVs or increased sensitivity may yield more conclusive data,. However, it is an inherent challenge for studies using biobank material to extract sufficient EV materials, given a limited amount of biomaterial available from biobanks. The EV-size distribution measured by NTA (Fig 3) also indicated differences between the cancer biobank samples and DBDS biobank samples, and direct comparisons between PD samples and OC samples should therefore be made in considerations of this. Although electron microscopy was performed to examine for the presence of EVs in the samples, samples were not comprehensively assessed for contamination by cellular debris, such as nuclear or mitochondrial contaminants. Another limitation is that although EV arrays were performed in triplicate per array per patient, individual patients were not measured more than once, as per assay protocol. Moreover, EV array data were not validated using other methods, such as immunoblotting or mass spectrometry. It is a further limitation that the miRNA discovery study was performed only in OC vs. B samples, and it could have been relevant to also perform miRNA Taqman Arrays for the Ctrl and PD groups.

In conclusion, we thoroughly analyzed EVs in ovarian cancer, women with benign tumors, healthy blood donors and blood donors with incident OC. EV CD9, CD151 and CD81 showed promising results in differentiating OC from healthy controls, while EV miRNAs were unable to distinguish groups from each other. Further studies should focus on larger groups and test EV CD9, CD151 and CD81 in conjunction with other risk factors and well-established biomarkers like CA125 or HE4. However, more research is required, focusing on preanalytical variables such as storage temperature and EV sample preparation.

## Supporting information

S1 Table EV Array antibody overview. The antibodies used for EV Array, and the criteria for selection.
(DOCX)

S2 Table Oligonucleotide sequences. Oligo sequences of the miRNAs tested in the technical replication and validation study.
(DOCX)

S3 Table microRNA array data underlying Fig 5, displaying for each detected EV microRNA the fold change between ovarian cancer and benign tumor, the t-tes p-value and adjusted p-values. (XLSX)

S1 Fig Selected samples were imaged by transmission electron microscopy. Arrows point to EVs.
(TIF)

S2 Fig Additional EV protein markers based on EV array identified in the four groups: OC, B, Ctrl, and PD, depicted as relative mean intensity with standard deviation (SD) (a.u.). A. Mucin16, B. CEA, C. MUC1, D. HER3. Statistical comparisons between groups were made using one-way ANOVA on logarithmic transformed data with Tukey’s test for multiple comparisons.
(TIF)

## Abbreviations

miRNA: microRNA
OC: ovarian cancer
B: benign tumor
PD: prediagnostic
EV: extracellular vesicle
DLS: dynamic light scattering
NTA: nanoparticle tracking analysis
nFCM: Nano Flow cytometry
