# Supplementary material for: Characterization of small extracellular vesicles from ovarian cancer patients and pre-diagnostic patient samples: Evidence from the Danish blood donor study
Source: PLoS One. 2025 May 15;20(5):e0323529. doi: 10.1371/journal.pone.0323529 (PMC12080785; doi:10.1371/journal.pone.0323529)
Supplement: S1 Table — The antibodies used for EV Array, and the criteria for selection. (DOCX) [file pone.0323529.s001.docx]

| **Antibody** | **Producent** | **Clone** | **Catalog no** | **Selection criteria** |
| --- | --- | --- | --- | --- |
| Annexin V | R & D Systems | - | AF399 | A marker for apoptosis |
| CD9 | Ancell | SN4/C3-3A2 | 156-020 | A common exosome marker |
| CD63 | Biorad | MEM-259 | MCA2142 | Is often used as a marker for multivesicular bodies, and extracellular vesicles |
| CEA | R & D Systems | 487609 | MAB41281 | It is a tumor marker, used for monitoring the treatment of OC pts. |
| CD81 | Ancell | 1.3.3.22 | 302-020 | A common vesicle marker |
| Mucin16 | Santa Cruz Bio | X306 | Sc-52095 | CA125 |
| TSG101 | Abnova | - | H00007251-mo1 | Exosome marker |
| CA19-9 | LS Bio | - | LS-B5680 | Cancer antigen tumor marker used for OC, pancreatic and intestinal cancer. |
| Alix | Biolegend | 3A9 | 634501 | Exosome marker |
| CD151 | R & D Systems | 210127 | MAB1884 | Often seen in relation to cancer |
| Tspan8 | R & D Systems | 458811 | MAB4734 | Often seen in relation to cancer |
| BRCA1 | Santa Cruz | D-9 | sc-6954 | Breast cancer susceptibility gene |
| LAMP2 | R & D Systems |  | MAB6228 | Lysosome and late endosome marker |
| CAIX | Abcam | 2D3 | ab107257 | Possible prognostic marker |
| CA12 | LS Bio | - | LS-C35272 | Possible prognostic marker |
| CD36 | Santa Cruz | SMɸ | sc-7309 | Is expressed on OC cells |
| ASGR1/ASGPR1 | R&D Systems | 950216 | MAB4394 | Tissue marker – liver |
| FATP4 | R&D Systems | 322142 | MAB3650 | Tissue marker – fat |
| MUC1 | R & D Systems | 604804 | MAB6298 | Overexpression identified as linked to e.g., OC |
| HER4/ErbB 4 | Abcam | H4.77.16 | ab3104 | Human epidermal growth factor receptor 4, used in OC and BC |
| PLAP | Santa Cruz Bio | 8B6 | Sc-47691 | Placental alkaline phosphatase, is a tumor marker for e.g., OC. |
| Integrin β6 | Santa Cruz | C-19 | sc-293194 | Is linked to metastasizing in OC |
