# Supplementary material for: Characterization of small extracellular vesicles from ovarian cancer patients and pre-diagnostic patient samples: Evidence from the Danish blood donor study
Source: PLoS One. 2025 May 15;20(5):e0323529. doi: 10.1371/journal.pone.0323529 (PMC12080785; doi:10.1371/journal.pone.0323529)
Supplement: S2 Table — Oligo sequences of the miRNAs tested in the technical replication and validation study. (DOCX) [file pone.0323529.s002.docx]

| **miRNA name** | **Oligonucleotide sequence (5’ to 3’)** |
| --- | --- |
| hsa-miR-150-5p RT | CTCAACTGGTGTCGTGGAGTCGGCAATTCAGTTGAG CTGTCCCC |
| hsa-miR-150-5p FW | ACACTCCAGCTGGG CTGGTACAGGCCTGG |
| hsa-miR-342-3p RT | CTCAACTGGTGTCGTGGAGTCGGCAATTCAGTTGAGACGGGTGCG |
| hsa-miR-342-3p FW | ACACTCCAGCTGGG TCTCACACAGAAATCG |
| hsa-miR-485-3p RT | CTCAACTGGTGTCGTGGAGTCGGCAATTCAGTTGAG AGAGAGGA |
| hsa-miR-485-3p FW | ACACTCCAGCTGGG GTCATACACGGCTCT |
| hsa-miR-29a RT | CTCAACTGGTGTCGTGGAGTCGGCAATTCAGTTGAGTAACCGAT |
| hsa-miR-29a FW | AC ACT CCA GCT GGG TAG CAC CAT CTG AAA T |
| hsa-miR-26a RT | CTCAACTGGTGTCGTGGAGTCGGCAATTCAGTTGAG AGCCTATC |
| hsa-miR-26a FW | ACACTCCAGCTGGG TTCAAGTAATCCAGG |
| hsa-miR-328-3p RT | CTCAACTGGTGTCGTGGAGTCGGCAATTCAGTTGAGACGGAAGGG |
| hsa-miR-328-3p FW | ACACTCCAGCTGGG CTGGCCCTCTCTGCC |
| hsa-miR-106b-5p RT | CTCAACTGGTGTCGTGGAGTCGGCAATTCAGTTGAGATCTGCACT |
| hsa-miR-106b-5p FW | ACACTCCAGCTGGG TAAAGTGCTGACAG |
| hsa-miR-146b-5p RT | CTCAACTGGTGTCGTGGAGTCGGCAATTCAGTTGAGCAGCCTATG |
| hsa-miR-146b-5p FW | ACACTCCAGCTGGG TGAGAACTGAATTCCA |
| hsa-miR-21 RT | CTCAACTGGTGTCGTGGAGTCGGCAATTCAGTTGAGTCAACATC |
| hsa-miR-21 FW | AC ACT CCA GCT GGG TAG CTT ATC AGA CTG A |
| U6 FW | AACGCTTCACGAATTTGCGT |
| U6 RT | CTC GCT TCG GCA GCA CA |
| FW hsa-mR-486-5p | ACACTCCAGCTGGG TCCTGTACTGAGCTG |
| RT hsa-miR-486-5p | CTCAACTGGTGTCGTGGAGTCGGCAATTCAGTTGAGCTCGGGGC |
