# Supplementary figures and images for: Characterization of small extracellular vesicles from ovarian cancer patients and pre-diagnostic patient samples: Evidence from the Danish blood donor study
Source: PLoS One. 2025 May 15;20(5):e0323529. doi: 10.1371/journal.pone.0323529 (PMC12080785; doi:10.1371/journal.pone.0323529)

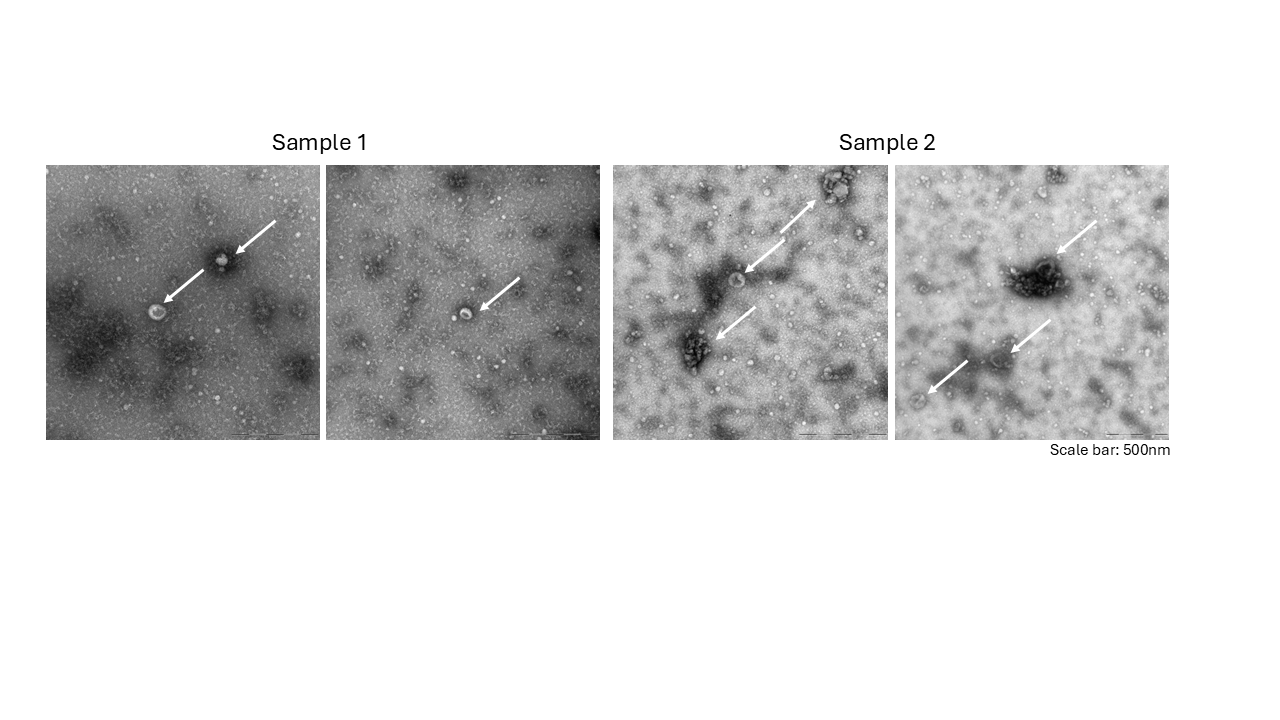

Supplement: S1 Fig — Arrows point to EVs. (TIF) [file pone.0323529.s004.tif]

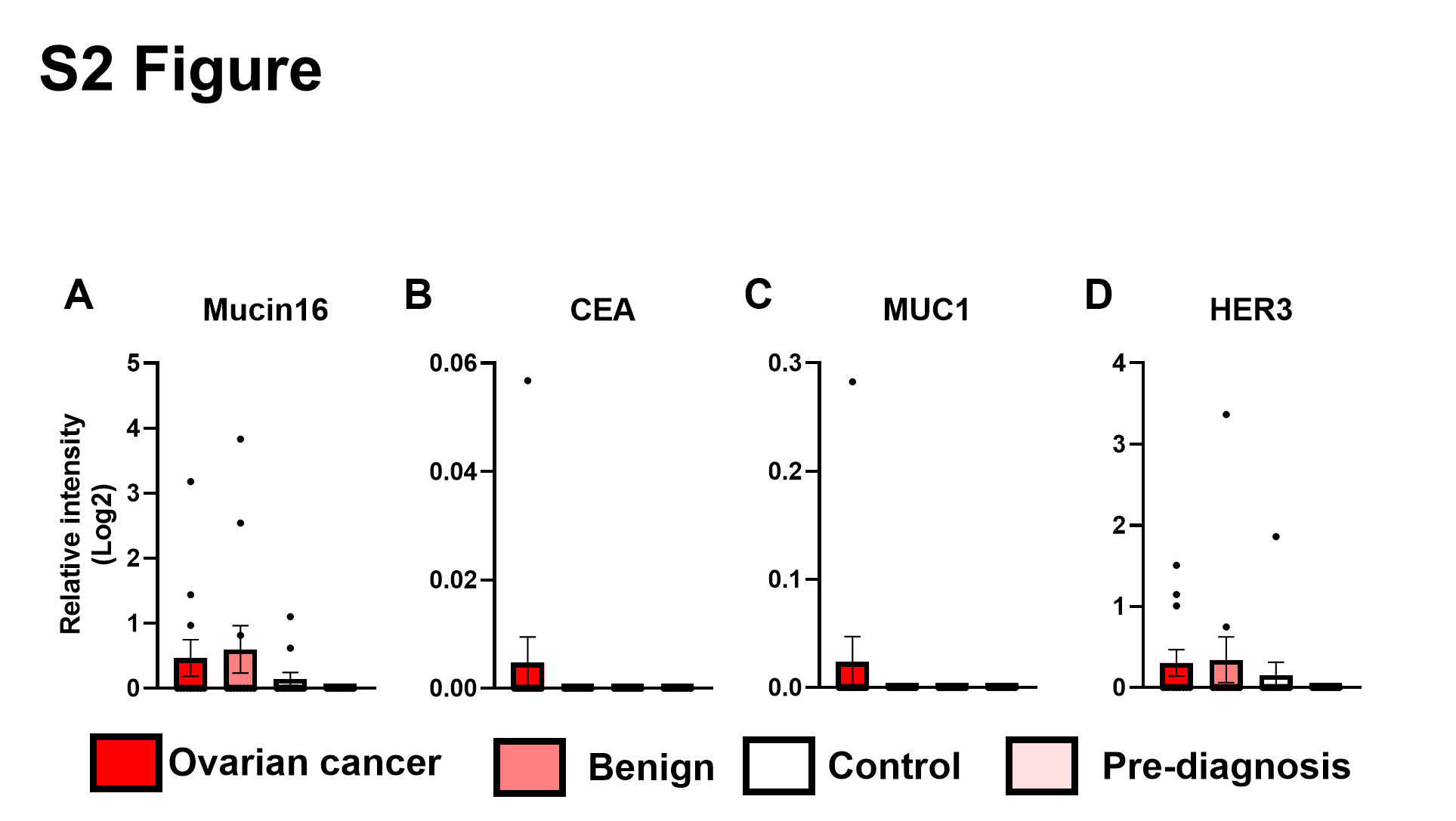

Supplement: S2 Fig — A. Mucin16, B. CEA, C. MUC1, D. HER3. Statistical comparisons between groups were made using one-way ANOVA on logarithmic transformed data with Tukey’s test for multiple comparisons. (TIF) [file pone.0323529.s005.tif]
